# Botulinum Toxin Type A for the Treatment of Auriculotemporal Neuralgia—A Case Series

**DOI:** 10.3390/toxins15040274

**Published:** 2023-04-06

**Authors:** Yan Tereshko, Enrico Belgrado, Christian Lettieri, Gian Luigi Gigli, Mariarosaria Valente

**Affiliations:** 1 Clinical Neurology Unit, Udine University Hospital, Piazzale Santa Maria della Misericordia 15, 33100 Udine, Italy; gianluigi.gigli@uniud.it (G.L.G.); mariarosaria.valente@uniud.it (M.V.); 2 Neurology Unit, Udine University Hospital, Piazzale Santa Maria della Misericordia 15, 33100 Udine, Italy; enrico.belgrado@asufc.sanita.fvg.it (E.B.); christian.lettieri@asufc.sanita.fvg.it (C.L.)

**Keywords:** auriculotemporal neuralgia, botulinum toxin, pain

## Abstract

Auriculotemporal neuralgia is a rare pain disorder in which anesthetic nerve blockade is usually effective but not always resolutive. Botulinum toxin type A has proven to be effective in treating neuropathic pain, and patients with auriculotemporal neuralgia could also benefit from this treatment. We described nine patients with auriculotemporal neuralgia treated with botulinum toxin type A in the territory of auriculotemporal nerve innervation. We compared the basal NRS and Penn facial pain scale scores with those obtained 1 month after BoNT/A injections. Both Penn facial pain scale (96.67 ± 24.61 vs. 45.11 ± 36.70, *p* 0.004; mean reduction 52.57 ± 36.50) and NRS scores (8.11 ± 1.27 vs. 4.22 ± 2.95, *p* 0.009; mean reduction 3.89 ± 2.52) improved significantly at one month after treatment. The mean duration of the effect of BoNT/A on pain was 95.00 ± 53.03 days and no adverse effects were reported.

## 1. Introduction

The auriculotemporal nerve (ATn) arises from the posterior division of the mandibular nerve, passes through the *foramen ovale*, and travels beneath the *pterygoideus externus* muscle to the medial aspect of the neck of the mandible. It continues upward, beneath the parotid gland, adjacent to the *superficial temporal artery*, and between the ear and the condyle of the mandible. It continues, beyond the parotid gland, over the *zygomatic arch*, and in front of the *temporomandibular joint* (TMJ). The auriculotemporal nerve (ATn) then pierces the *temporalis* muscle and divides into five branches: nerve to the external auditory meatus (innervating the tragus as well), anterior auricular nerve (which innervates the anterior part of the auricle), parotid branches, articular branches (innervating the posterior aspect of the TMJ), and the superficial temporal nerve (it innervates the skin of the temporal region) [1,2,3]. Despite this branch division, the ATn has a wide anatomical variety presenting only one branch in about fifty percent of specimens [4]. The auriculotemporal nerve provides cutaneous sensory innervation over the external acoustic meatus, the temporal cutis and the posterior region of the temple, the anterior region of the ear and the tragus, the parotid gland, the external tympanic membrane, and the TMJ [5,6]. There is evidence that the auriculotemporal nerve, or its branches, communicates with the facial nerve, the *zygomaticotemporal* nerve, the *lesser occipital* nerve, and/or the *greater occipital* nerve [3,5,7,8]. These communications could explain the radiating pain involving the occipital, retro-orbital, and retro-auricular regions, as well as facial muscle pain. In addition to sensory innervation, the ATn carries autonomic fibers from the *middle meningeal artery plexus* and *lesser petrosal nerve* to the parotid gland.

Based on its anatomical distribution, pain arising from this branch may also be perceived in the temporal region, TMJ, in the parotid, retro-auricular and retro-orbital region. 

Auriculotemporal neuralgia (ATN) is a rare facial pain disorder involving the auriculotemporal nerve (ATn) characterized by unilateral and side-locked pain localized in the temporal scalp, auricular region, temporomandibular joint, and/or parotid region. It is a rare and unusual facial pain disorder that occurs with a frequency of 0.4% in tertiary headache outpatient clinics [9]. The pain typically occurs as paroxysmal attacks in the preauricular and temple regions with the possible radiation of pain in the occipital, retro-orbital, and temporal scalp regions. Damarjian described a total of 34 patients with ATN in 1970 [10]. Speciali et al. described six patients with ATN in 2005 [9]; all the patients were female and all of them had unilateral pain which worsened with palpation over the preauricular region, with secondary pain radiation toward the mandible in all cases, and toward the occipital region in four cases. The presence of concomitant background pain is not uncommon [10,11,12]. Ruiz et al. reported eight patients with ATN: two patients had only background pain, two patients had both background pain and paroxysmal stabbing pain, while the last four patients only had paroxysmal pain (two of them had stabbing pain, one had shock-like pain and one had throbbing pain); exacerbation pain lasted from 2 s to 30 min and was mainly localized in the temporal, preauricular, and periauricular regions, but there was also hemicraneal and lateral neck localization and/or pain radiation. In 2015, Kosminsky et al. described another case of ATN [12]; they also reported that they treated other five patients with ATN (six patients in total and five of them had persistent pain).

The quality of pain is frequently throbbing, possibly due to the proximity to the temporal artery, but could also be lancinating, stabbing, or shock-like, and the intensity is moderate to severe. The symptoms could be triggered by chewing, talking, jaw traumas, gustatory stimuli, facial tactile stimuli, or pressure over the preauricular region. Tenderness and allodynia over the preauricular region are frequent features [3]. The entrapment of the auriculotemporal nerve (ATn) at the level of the *pterygoideus externus* muscle due to muscle spasm or inflammation, the wrapping around the *superficial temporal artery*, or the compression at the level of fascia bands in the preauricular area, were proposed to be possible causes of the issue [2,13,14]. The ATn could also be entrapped between the *pterygoideus externus* and *pterygoideus internus* muscles [15]; moreover, the mandibular overclosure due to a lack of posterior teeth was also described [16]. Other factors associated with ATN are mandibular fracture, TMJ synovial cyst, and TMJ pathology. Although ATN is not reported in the third edition of the International Classification of Headaches Disorders (ICHD-III), Ruiz et al. proposed the diagnostic criteria for this disorder [11,17]. The anesthetic nerve blockade of the auriculotemporal nerve (ATn) is considered both therapeutic and diagnostic; the occurrence of pain relief after the procedure is one of the diagnostic criteria and its effect could even last several years [9,10], however, is not always resolutive.

Damarjian performed from one to fourteen local anesthetic nerve blockades in ATN patients describing a recurrence rate of 30% after 1–2 years [10]. Carbamazepine, trigeminal ganglionar block, and gabapentin have been used in some case series with some positive results [9,11]. 

The literature is scarce with regard to the therapy of refractory cases, but a recent article reported one case of successfully treated refractory ATN with botulinum toxin type A (BoNT/A) [18]. Botulinum toxin (BoNT) is produced by clostridium botulinum and there are seven different serotypes (A, B, C, D, E, F, and G), but only serotypes A and B are currently used in clinical practice. BoNT is used in the treatment of focal and segmental dystonias, spasticity, and strabismus due to its ability to induce flaccid paralysis [19]. BoNT/A has also been demonstrated to be effective in neuropathic pain and neuralgias, with a level A of evidence in trigeminal neuralgia, post-herpetic neuralgia, and post-traumatic neuralgia [20]. Hereby, we report our experience in treating the patients with ATN, who addressed our headache outpatient clinic for an alternative relief, with BoNT/A. 

## 2. Results

Altogether, data from nine patients were collected (five males and four females; mean age 48.77 ± 13.22). The mean duration of the ATN was 10.38 ± 12.21 months. The pain was unilateral in all cases (six left-sided and three right-sided) and it was localized in the ATn innervation region. Radiating pain frequently involved the retro-orbital, occipital, or mandibular region; patients three and four referred infrequent ipsilateral cervical and/or neck radiating pain. Pressure over the temporal fossa and preauricular area caused pain exacerbation in all the patients; seven out of nine had allodynia in the ipsilateral temporal region, while one had hypoesthesia (patient 7). Seven patients had dull background pain in the territory of the auriculotemporal nerve with daily paroxysmal exacerbations, while two had paroxysmal pain only. The pain was mainly described as stabbing, throbbing, or shock-like; however, one patient described the pain as burning. Six patients were on medications for pain although with minimal or no benefit. Anesthetic nerve blockade of the auriculotemporal nerve with 1–2 mL of 0.5% bupivacaine determined complete pain relief almost immediately, but only for a short period (from 1 day to 10 days). See Table 1 for details.

The ATn anesthetic nerve blockade was repeated seven times in patient 9 and five times in patient 8; the other patients were treated two times before BoNT/A therapy. Each procedure was able to abolish pain in every patient and the effect had a similar duration; the interval time between one anesthetic block and another was one week. No adverse effects were reported after this procedure. In our cohort, the mean dose of BoNT/A was 60.56 ± 19.44 U. The effect of BoNT/A started 7–10 days after the injections. Three patients (patients 1, 3, and 4) had a prolonged response (5–6 months duration) with complete pain relief occurring 3 months after the treatment; four patients (patients 2, 5, 6, and 9) showed partial pain relief which lasted for 3 months, while the last two patients (patients 7 and 8) had only a poor response with a short duration (30–45 days). Detailed results are summarized in Table 2. Overall, both PFPS (96.67 ± 24.61 vs. 45.11 ± 36.70, *p* 0.004; mean reduction 52.57 ± 36.50) and NRS scores (8.11 ± 1.27 vs. 4.22 ± 2.95, *p* 0.009; mean reduction 3.89 ± 2.52) improved significantly at one month after treatment. The mean duration of the effect of BoNT/A on pain was 95.00 ± 53.03 days. No adverse effects were observed or reported.

**Figure 1:**
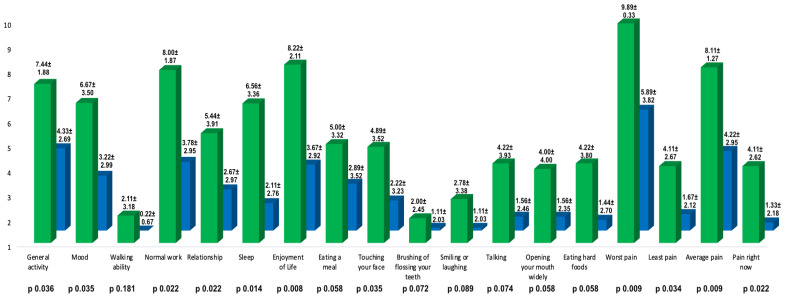
Means (expressed as number ± standard deviation) of every item of the PFPS at the baseline and 1 month after the BoNT/A injections. The green bars indicate the means of the items of PFPS at the baseline while the blue bars indicate the items 1 month after BoNT/A treatment. The first 14 items describe, using a score from 0 to 10, how much the pain has interfered with those activities in the past week; the last four items (worst pain, least pain, average pain, and pain right now) describe the pain (score from 0 to 10) in the past week. A paired *t*-test or a Wilcoxon test was used for the comparison; significance was determined with a *p*-value of 0.05.

The analysis of every item of the Penn facial pain scale (PFPS) showed that BoNT/A treatment significantly reduced the interference of ATN pain in general activity (*p* 0.036), mood (*p* 0.035), normal work (*p* 0.022), relationship with other people (*p* 0.022), sleep (*p* 0.014), enjoyment of life (*p* 0.008), and touching the face (*p* 0.035). There was also a reduction of the “worst pain” (*p* 0.009), “least pain” (*p* 0.034), “average pain” (*p* 0.034), and “pain right now” (*p* 0.022) items (Figure 1).

## 3. Discussion

The differential diagnosis mainly arises with temporomandibular disorders (TMD), migraine, continuous hemicrania, otitis, affection of the parotid gland, temporal arteritis, odontalgia, nummular headache, atypical facial pain, and trigeminal neuralgia (TN). TN is characterized by recurrent attacks of lancinating or stabbing pain in the dermatomal distribution of the trigeminal nerve, sometimes associated with a continuous background facial pain; the pain usually affects the V2 and V3 distributions while only less than 5% of patients suffer from pain in the V1 distribution. Autonomic symptoms such as conjunctival injection or tearing, miosis, ptosis, sweating, and clogged nose can occur with trigeminal neuralgia on the same side as the pain. The most frequent triggers in TN are mastication, brushing teeth, speaking, touching the site of pain, and cold wind exposure. 

Antiseizure drugs and tricyclic antidepressants are effective treatments, in particular carbamazepine, which is the first-line therapy for TN. In refractory cases, surgical treatment such as microvascular decompression, radiofrequency, or thermocoagulation can be performed [21]. Alternatively, in ATN, the anesthetic blockade is the most effective treatment, although carbamazepine, gabapentin, and trigeminal ganglionar block were reported to be effective in some cases [9,10,11]. In our cohort, we excluded trigeminal neuralgia since the pain was localized in the auriculotemporal innervation region, such as the temporal fossa and preauricular area, had different quality and modalities of presentation, and ATn anesthetic nerve blockade was able to completely abolish the pain. In only one case, we suspected temporal arteritis, but this diagnosis was excluded due to normal superficial temporal artery Doppler ultrasound, normal erythrocyte sedimentation rate (ESR), C-reactive protein (CRP), and a normal complete blood count; autoantibodies such as antinuclear antibodies, ANCAs, and rheumatoid factor were also negative. We excluded nummular headache because our patients were not able to delimitate a well-circumscribed coin or ellipsoidal-shaped area; normal TMJ CT or MRI and physical examination excluded TMD. ATN pain characteristics and the occurrence of nausea and vomiting could meet the criteria for episodic or chronic migraine, according to ICHD-3 [17]; in our case series, we excluded migraine for the following reasons: the pain was side-locked and was localized in the territory of the auriculotemporal nerve, the involvement of the other side was never described by the patients, radiating pain in the occipital or retro-orbital regions was common but not always present during paroxysmal pain attacks, auriculotemporal nerve anesthetic blockade was able to completely abolish pain after a few minutes, the pain quality was not fully consistent with migraine, photophobia/phonophobia/osmophobia were not present, the pain was triggered or exacerbated by pressure only over the ipsilateral preauricular region, and the duration of pain paroxysms was variable, lasting from a few seconds to 30 min. Hemicrania continua, otitis, odontalgia, and atypical facial pain were also ruled out.

In our tertiary care center, referring from a large area, the prevalence of ATN among headache patients in our outpatient clinic was 0.23% and the mean age of onset was 48.22 ± 13.01 years, in accordance with the previous literature [9,10,11]. Although this disorder is reported to be more common among women [9,10,11], our cohort was composed mostly of men (55.56%). All the patients fulfilled the criteria for ATN according to Ruiz et al. [11] and the diagnosis was supported by complete pain relief following anesthetic nerve blockade. Nevertheless, the duration of the effect was short (24 h–10 days). ATN is a rare disorder and treatments proposed in the literature are not evidence-based; therefore, most clinicians prescribe medications used in other forms of neuralgia, such as gabapentin or carbamazepine [9,10,11,12]; in our cohort, the patients that were on medications had no significant pain relief with these drugs. Treatment with BoNT/A injections had been previously reported in only one case of ATN with satisfactory results [18]. Moreover, it was proven to be effective in other cranial neuralgias, such as trigeminal and occipital neuralgia [22,23]. BoNT/A modulates neuropathic pain by inhibiting the release of the neurotransmitters involved in central and peripheral sensitization (CGRP and substance P) [24,25,26,27]. Moreover, BoNT/A has selectivity for afferent nerve fibers expressing TRPV1, which are only involved in pain and mechanical stimulation [28,29], selectivity for interfering with TRPV1 expression on the plasma membrane of peripheral sensory fibers, for neuronal ganglia and also for central nervous system neurons [30,31]. This phenomenon is probably due to the retrograde axonal and trans-synaptic transport of BoNT/A [31,32,33]. Moreover, within the central nervous system, it was demonstrated that BoNT/A also attenuates microglia activation and enhances brainstem and dorsal horn inhibitory endogenous opioid and GABA systems [34,35,36,37]. The evidence of BoNT/A safety and efficacy in neuropathic pain and the lack of effective and evidence-based treatments in ATN, especially in refractory cases, convinced us to perform this treatment in our cohort of patients. In our case series, three patients had complete relief from pain for 5–6 months and four patients had a significant attenuation of pain for 3 months. Only two patients had a minimal response to the treatment. Although our data are encouraging, the retrospective uncontrolled design of the study does not consent to exclude a concomitant placebo effect of an unknown degree. Another limitation of our study is characterized by the use of diagnostic criteria proposed by Ruiz et al. in 2016 [11] for the diagnosis of auriculotemporal neuralgia. Thus, there is a need for official diagnostic criteria to better characterize this issue and define its clinical features.

## 4. Conclusions

BoNT/A could be a safe and effective alternative treatment in cases of auriculotemporal neuralgia. Further studies, specifically placebo-controlled studies, are needed to confirm the efficacy and safety of this procedure in the treatment of ATN.

## 5. Materials and Methods

This is a retrospective longitudinal single-center study. We retrospectively collected the data from 9 patients affected by ATN seen in our tertiary headache outpatient clinic from January 2017 to September 2022. Before the diagnosis of ATN, every patient performed an extensive neurological examination, neuroimaging (brain and TMJ MRI or CT), and blood work-up to exclude secondary causes. Temporo-mandibular joint function and mobility and peripheral cranial nerve tenderness were evaluated. Diagnosis of ATN was performed according to the criteria suggested by Ruiz et al. [11].

**Figure 2:**
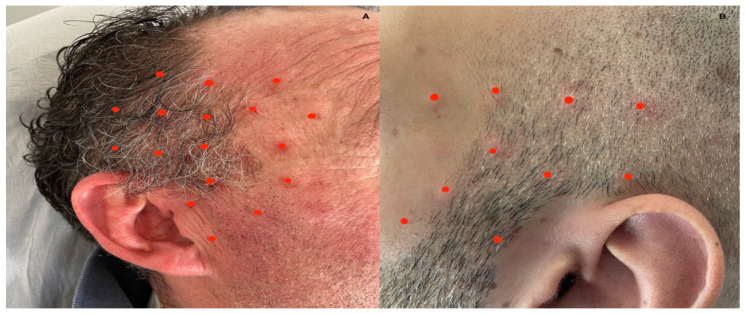
(**A**) shows the injection sites (red dots) treated in patient 9 while (**B**) shows the injection sites treated with 50 U in patient 2; we performed every treatment with 5 U injections of BoNT/A per site, 100 U/mL BoNT/A dilution with 0.9% sodium chloride.

The diagnostic and therapeutic anesthetic nerve blockade of the auriculotemporal nerve was performed with the patient lying supine; the auriculotemporal nerve was located in the temporal fossa and slightly anterior to the superficial temporal artery, at the apex of the isosceles triangle with the base formed by the line connecting the tragus with the lateral cantus of the eye [3]. The temporal artery was identified through palpation to avoid accidental injections; the needle was inserted at a 45° angle in the cephalad direction with a 5–10 mm depth. An amount of 1–2 mL of 0.5% bupivacaine was used for local anesthesia during this procedure. The anesthetic blockade determined complete pain relief in all the patients although its duration was short (24 h–10 days). Since there are no evidence-based studies regarding ATN therapy except for anesthetic nerve blockade, we proposed BoNT/A treatment as an alternative therapy for ATN. BoNT/A was considered due to its positive effect in improving pain in neuralgias such as trigeminal neuralgia and occipital neuralgia [38]. In our cohort of patients, BoNT/A injections were performed 2–3 weeks after the anesthetic blockade; this decision was to ascertain the effect of the anesthetic blockade was over. A 100 IU vial of BoNT/A was diluted with 1 mL 0.9% sodium chloride; every patient was treated with BoNT/A in the territory of the auriculotemporal nerve with a variable total amount of units (5 U per site); the total dose was decided based on the severity and distribution of the symptoms of each patient. Treatment was performed with subcutaneous injections of botulinum toxin type A (BOTOX^®^) using a 30-gauge 0.30 × 8 mm needle. When performing the subcutaneous injections, we grasped the area of the skin surrounding the injection site to separate it from the muscular layer and then we thrust the needle with an injection angle of 30–45° into the subcutaneous tissue (Figure 2).

Clinical examinations were completed by the administration of the Penn facial pain scale (PFPS) [39], numeric rating scale (NRS) [40] (performed at 1 month and after 3 months of BoNT/A treatment), and patients’ global impression of change (PGIC) [41] scale (assessed 3 months after BoNT/A injections). The NRS is an assessment tool for pain severity using a score that ranges from 0 (no pain) to 10 (worst pain imaginable) [40]. The Penn facial pain scale assesses the facial pain interference with the activities of daily living with particular attention to facial-related activities; this scale also evaluates facial pain intensity (worst pain, least pain, average pain, pain right now) using the NRS. We compared the basal NRS and PFPS scores with those obtained 1 month after BoNT/A injections. The study was conducted in accordance with the Declaration of Helsinki and was approved by the local ethics committee (RIF. Prot IRB: 150/2022). The patients gave their informed consent for the treatment with anesthetic blockade and BoNT/A and for their images and other clinical information to be reported in the journal.

### Statistical Analysis

A descriptive analysis of the study population’s continuous variables was performed using mean ± standard deviation (SD). For the comparison of PFPS and NRS between baseline and 1 month after BoNT/A injections, we utilized a paired *t*-test, when the data had normal distribution, or a Wilcoxon test when the distribution of the data was not normal. A Shapiro–Wilk test was used to assess the normal distribution of data. All analyses were conducted using Stata/SE (version 15.1, StataCorp) for Mac OS. Statistical significance levels were set at *p* < 0.05.

## Figures and Tables

**Table 1:** Characteristics of ATN duration, side, radiating pain sites, pain quality, triggers, background pain, paroxysmal pain, concomitant treatment, and the response to anesthetic blockade of the auricular nerve in our cohort of patients. The time interval between one anesthetic block and another was one week. Legend: NRS, numeric rating scale; QD (quaque die), once a day; TID (ter in die), three times a day; ATn, auriculotemporal nerve; ATN, auriculotemporal neuralgia.

Patient	Sex/Age	ATN Duration	ATN Side	ATN Localization	Possible Radiating Pain	Pain Quality	Pain Exacerbation or Triggers	Background Pain	Paroxysmal Pain	Concomitant Treatment	Anesthetic Blockade: Dose, Anesthetic, Onset, Duration, Number of Procedures
1	F/51	7 months	Left	Preauricular Temporal	Retro-orbital Occipital	Throbbing and stabbing	Talking for long periods. Pressure over ATn.	-	NRS 5/10 1/day 5–30 min	None	1 mL bupivacaine: 10 min after there was complete resolution of pain for 1 day; 2 procedures.
2	M/30	1 month	Left	Temporal	Retro-orbital	Stabbing	Talking and chewing. Pressure over ATn.	NRS 6/10	NRS 8/10 1–2/day 15–30 min	Ibuprofen 600 mg TID and paracetamol 1000 mg TID	1 mL bupivacaine: 5 min after there was complete resolution of pain for 1 day; 2 procedures.
3	M/73	5 months	Right	Temporal Supra-auricular	Occipital	Burning and shock-like	Pressure over ATn.	NRS 4/10	NRS 8/10 15/day 10–15 min	None	2 mL bupivacaine: 5 min after there was complete resolution of pain for 10 days; 2 procedures.
4	M/45	2 months	Right	Preauricular Temporal	Occipital Retro-orbital	Shock-like and burning	Pressure over ATn.	NRS 2/10	NRS 8/10 10–18/day 30 s–5 min	Carbamazepine 800 mg QD, Pregabalin 150 mg QD and indometacin 200 mg QD	2 mL bupivacaine: 5 min after there was complete resolution of pain for 1 day; 2 procedures.
5	F/35	2 weeks	Left	Temporal	Retro-orbital	Throbbing Stabbing	Pressure over ATn.	NRS 2/10	NRS 9/10 20/day 2–10 s	Pregabalin 50 mg QD	1 mL bupivacaine: 5 min after there was complete resolution of pain for 3 days; 2 procedures.
6	F/47	2 years	Left	Preauricular Temporal	Occipital	Throbbing and stabbing	Pressure over ATn.	NRS 5/10	NRS 9/10 3–4/day 5–20 min	Venlafaxin 75 mg QD	1 mL bupivacaine: 5 min after there was complete resolution of pain for 1 day; 2 procedures.
7	M/66	3 years	Left	Temporal	Retro-orbital	Stabbing	Pressure over ATn.	-	NRS 9/10 1/day 10 min–30 min	Pregabalin 150 mg QD, tapentadol 150 mg QD	1 mL bupivacaine: 5 min after there was complete resolution of pain for 2 days; 2 procedures.
8	F/45	4 months	Left	Preauricular Temporal	Mandibular	Stabbing and shock-like	Wide opening of the mouth. Pressure over ATn.	NRS 4/10	NRS 7/10 3–4/day 30–60 s	Pregabalin 150 mg QD, carbamazepine 600 mg QD	1.5 mL bupivacaine: 5 min after there was complete resolution of pain for 2 days; 7 procedures.
9	M/49	14 months	Right	Temporal	Occipital	Throbbing and stabbing	Pressure over ATn.	NRS 3/10	NRS 8/10 3–4/day 20–40 min	None	1 mL bupivacaine: 5 min after there was complete resolution of pain for 2 days; 5 procedures.

**Table 2:** Therapeutic characteristics of BoNT/A treatment in our cohort. Legend: BoNT/A, botulinum toxin type A; PGIC, patient’s global impression of change; PFPS, Penn facial pain scale; NRS, numeric rating scale.

Patient	BoNT/A Units	Baseline PFPS	1 Month PFPS	Baseline Mean NRS	1-Month Mean NRS	3 Months Mean NRS	3 Months PGIC	Onset of the BoNT/A Effect	Duration of BoNT/A Effect on Pain
1	40 U	108	33	5	2	0	7/7	10 days	6 months
2	50 U	85	58	8	6	8	5/7	10 days	2 months
3	70 U	82	0	8	0	0	7/7	7 days	5 months
4	80 U	119	0	8	0	0	7/7	7 days	5 months
5	80 U	65	33	9	5	6	6/7	7 days	3 months
6	60 U	147	102	9	6	7	5/7	10 days	3 months
7	30 U	102	82	9	7	9	4/7	10 days	45 days
8	50 U	80	76	9	8	9	4/7	14 days	1 month
9	85 U	91	21	8	4	5	5/7	10 days	3 months

## Data Availability

Not applicable.
